# A Novel System for Correction of Relative Angular Displacement between Airborne Platform and UAV in Target Localization

**DOI:** 10.3390/s17030510

**Published:** 2017-03-04

**Authors:** Chenglong Liu, Jinghong Liu, Yueming Song, Huaidan Liang

**Affiliations:** 1 Changchun Institute of Optics, Fine Mechanics and Physics, Chinese Academy of Sciences, Changchun 130033, China; liuchenglong14@mails.ucas.ac.cn (C.L.); songyueming@ciomp.ac.cn (Y.S.); lianghuaidan14@mails.ucas.ac.cn (H.L.); 2 University of Chinese Academy of Sciences, Beijing 100049, China

**Keywords:** UAV, target localization, shock absorber, angular displacement, image registration, coordinate transformation

## Abstract

This paper provides a system and method for correction of relative angular displacements between an Unmanned Aerial Vehicle (UAV) and its onboard strap-down photoelectric platform to improve localization accuracy. Because the angular displacements have an influence on the final accuracy, by attaching a measuring system to the platform, the texture image of platform base bulkhead can be collected in a real-time manner. Through the image registration, the displacement vector of the platform relative to its bulkhead can be calculated to further determine angular displacements. After being decomposed and superposed on the three attitude angles of the UAV, the angular displacements can reduce the coordinate transformation errors and thus improve the localization accuracy. Even a simple kind of method can improve the localization accuracy by 14.3%.

## 1. Introduction

Currently, enemy situation reconnaissance, target localization, directing and adjusting artillery fire, and other auxiliary functions are still the main UAV applications. With a very low safety risk [1], an operator can remotely operate an UAV to fly toward the target area in order to acquire the target's real-time image and location information, which can be sent to the control center for analysis and decision making by intelligence analysts and commanders. In several recent wars, UAV has played a key role in situations where it is used for real-time battlefield reconnaissance, for collecting and providing intelligence, and for providing accurate target information to facilitate firing. For civil use, such as search and rescue [2], target localization is also the important UAV applications.

The basic principle of current target localization is the *R-θ* (remove-angle) method [3,4,5,6,7], in which the distance (*R*) of the target relative to the UAV is determined by a laser rangefinder and the angle (*θ*) is determined by a series of sensors. Based on its own location (usually using the Earth coordinate system), the position of a target in the geodetic coordinate system can be obtained after a series of coordinate transformations. The common target localization process is usually done through the transformations among at least five coordinate systems, including the camera coordinate system C, UAV coordinate system (platform) B, UAV geographic coordinate system V, Earth-Centered Earth-Fixed coordinate system (ECEF) E (in line with the WGS-84 standard) and the geodetic coordinate system G (in line with the WGS-84 standard). The platform moves independently of the UAV, detects the target through rotating search, and outputs the azimuth and pitch information of the detected target relative to the UAV through continuous tracking [8]. Then these data are linked to the INS data of the UAV and converted into a geodetic coordinate system the same as the GPS standard.

Based on the combination of camera and laser range finder, this paper proposes a method to improve the accuracy of angles, which could improve the accuracy of single-point localization in real time. As the operating frequency of laser rangefinders in a single range-based localization task is quite low (usually at the Hz level), the system designed by this paper can be considered real-time provided that it can complete the ranging accuracy optimization in the range measurement period. In addition, this system takes up a small space, without a significant increase in cost. It can effectively improve the accuracy of one-time single-station localization and won’t interfere with most of the multi-times multi-station methods, so it is quite universal. At present, researchers have used various methods to analyze the localization error and improve the localization accuracy. Currently, some people are studying how to improve the single-aircraft single-point localization accuracy, while others are using the methods such as flight course planning and multi-aircraft localization to realize accuracy improvement. Most of the work done by current researchers fails to consider the deviation angle of the platform relative to the UAV. What has been considered, if possible, is only the addition of a parallel translation or the analysis of error influence, which, however, was seldom quantified. Redding [3] considered the influence of wind resistance, but the angular displacements caused by UAV attitude change were left out of consideration. The system in this paper can correct the angular displacements between the UAV and its platform when the cause of angular displacement error doesn't need to be known. The method proposed by Pachter et al. [9] also needs to know the target elevation in advance. Chiang et al. [10] improved the localization accuracy through setting the ground control point. Yue et al. [11] proposed the use of a height-based Steepest Descent Method for single-aircraft localization optimization, which, however, needs time and fails to meet the real-time requirement. While studying the single-aircraft localization, some people have begun to explore multi-aircraft localization. Morbidi et al. [12] proposed the method of active target tracking and joint localization based on an UAV fleet to estimate the target position through a Kalman filter. Qu et al. [13] proposed a joint localization method based on the azimuth angles among various UAVs. These methods, which are based on sensor data fusion, involve the complex issue of fleet route control [14,15,16] and need coordination among multiple UAVs, thus resulting in a higher hardware cost, more task time in most of the cases, and a greater risk in case of emergency. These exceptions apart, methods based on Kalman filter, Recursive Least Squares (RLS) filter, nonlinear filter [3,6,17,18] and methods based on video sequence [19,20,21,22,23,24] are also proposed to estimate the location. Most of these use the same aircraft at different time to improve the accuracy, but the optimization process requires time, that cannot meet the real-time requirement. On the other hand, the self-localization method integrating IMU into the platform involves the issue of size restriction. For a smaller IMU, its accuracy can’t meet the requirement easily so that an additional onboard IMU whose size is larger is needed, which will increase the load.

This paper is structured as follows: first, we build the target localization model. Then the principle and working process of the system designed in this paper are introduced in detail, followed by effectiveness analysis, experiments, simulation and verification, analysis of verification results, and finally a summary. 

## 2. Methodology 

**Figure 1:**
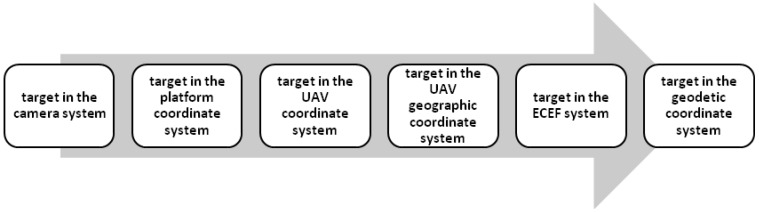
Localization process.

The core process of the target localization method adopted by this paper, is shown in Figure 1.

**Figure 2:**
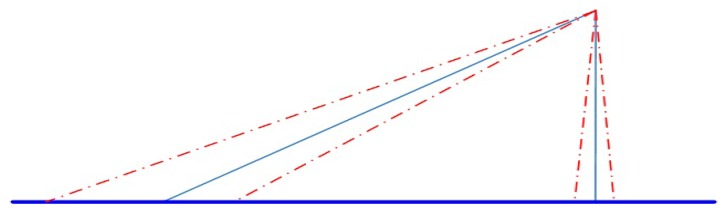
Different localization errors resulting from the same angle error.

Compared with most of the target localization models [25], the main advantage of this method is the separation of the platform coordinate system from UAV coordinate system [26]. The addition of transformational matrix has effectively reduced the directional error. This is more evident during the high-pitch big-slope reconnaissance. Take a large UAV whose rising limit is 8 km as an example, In the case of vertical down-view, the localization error brought by 1 mrad of angle error is only 8 m, but during the oblique-view reconnaissance, the slope distance can easily reach 30 km, where the localization error brought by the same angle error is as big as 30 m. As shown in Figure 2, the bigger the slope distances from the platform to the target, the higher the directional requirement. The specific localization process is as follows.

### 2.1. Transformation from Camera Coordinate System C to Platform Coordinate System P

**Figure 3:**
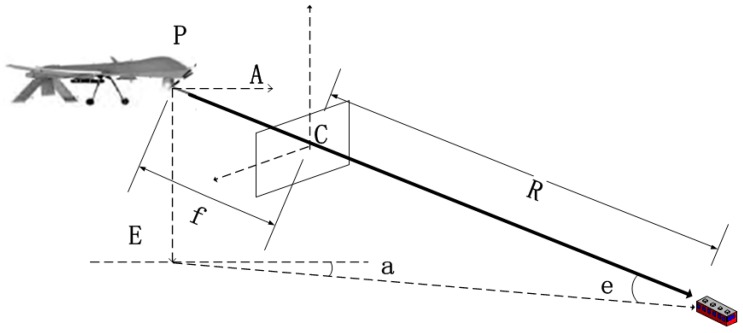
Relationship between camera coordinate system C and platform coordinate system P.

The relationship between the two coordinate systems is illustrated in Figure 3. 

The homogeneous coordinates of a target in the camera coordinate system are:

(1) $${\left[x_{c}\;y_{c}\;z_{c}\;1\right]}^{T}={\left[u\;v\;f\;1\right]}^{T},$$

where *u* and *v* are the target’s coordinates in the image (in pixel), and *f* is the current focal length of camera. Usually, when the UAV is detecting a target, the photoelectric platform will lock the detected target at the Field of View (FOV) center with multiple pixels and therefore the target can be considered at the image center. When the error inside camera coordinate system is ignored, the homogeneous coordinates of the target can be expressed as [0 0 *f* 1]*^T^*. A photoelectric platform is the camera carrier, which outputs the information on the angles a and e between Line of Sight (LOS) and the zero positions of two platform angles and measures the target distance R through a laser range finder. Since the platform uses the polar coordinate system, a coordinate transformation listed below is needed:

(2) $${\left[x_{p}\;y_{p}\;z_{p}\;1\right]}^{T}=R\times Q_{pc}{\left[0\;0\;f\;1\right]}^{T},$$

where *Q_pc_* is the conversion matrix from the camera coordinate system C to the platform coordinate system P.

(3) $$Q_{pc}=\left[\begin{array}{cccc} \cos a & 0 & \sin a & 0\\ 0 & 1 & 0 & 0\\ -\sin a & 0 & \cos a & 0\\ 0 & 0 & 0 & 1 \end{array}\right]\left[\begin{array}{cccc} 1 & 0 & 0 & 0\\ 0 & \cos e & -\sin e & 0\\ 0 & \sin e & \cos e & 0\\ 0 & 0 & 0 & 1 \end{array}\right].$$

### 2.2. Transformation from Platform Coordinate System P to UAV Coordinate System B

**Figure 4:**
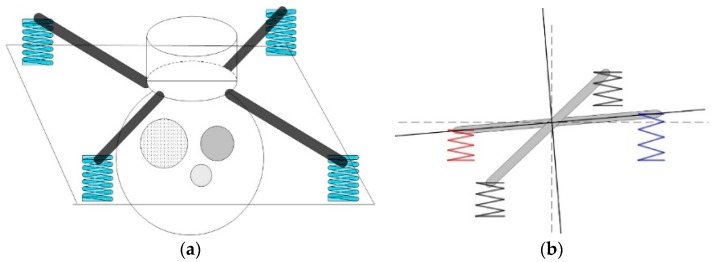
(**a**) Position of shock absorber; (**b**) Deformation of shock absorber.

This transformation process is the core of localization accuracy improvement over other methods [27]. Considering the demand for fast disconnection, the airborne platform is usually attached to the UAV in a strap-down manner. Most of the platforms have a shafting structure so that the onboard imaging systems (such as cameras and IR thermal imagers) can expand the reconnaissance field through rotation. Therefore, most of the platforms can be divided into two parts, namely the base and rotating part. The base is fixed to the UAV. The rotating part is linked, through shafting, to the base, putting the imaging system in motion to search for the target and locking the target to LOS via the servo system [28]. To improve the reconnaissance imaging quality, the base of an airborne platform often needs to be fixed to the UAV through a shock absorber, thus isolating part of high-frequency vibration and enhancing the stability of the platform itself, as show in Figure 4. However, the damping structure used by most of the shock absorber body is a flexible material, so in actual flight, the platform will produce a displacements relative to the UAV due to the influence of such factors as engine vibration, wind resistance, UAV attitude change and motion of platform. These displacements include monolithic translation, angular displacement and mixed displacement.

The target is usually locked in the center of the image (just like when you need to turn your head around to face a target and turn your eyes to the target so that you can look at it attentively) during flight. To determine the target angle we need to know the angle of the detected target relative to the platform, as output by the platform, and the angle of the platform itself relative to reference azimuth angle. Then the required angle can be obtained through transformation. For example, if a stone lies on the ground east-northeast of you, you need to know both the angle of the stone relative to your body and the angle of your body relative to the East or North. 

The Inertial Measurement Unit (IMU) can determine the UAV attitude angles. As stated above, the size of IMU will be larger if we need a high-precision one. Accordingly, the IMU in most of the cases is installed not on the platform, but in other UAV compartments through the rigid connection due to the limitation of platform size. Otherwise, the platform will be too large to be installed.

**Figure 5:**
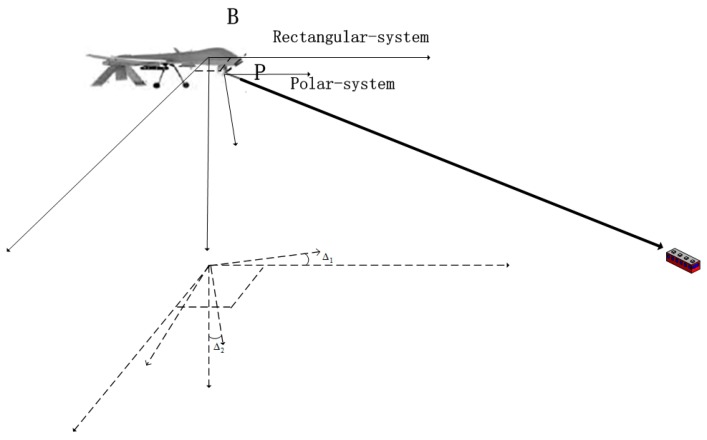
Relationship between platform coordinate system P and UAV coordinate system B.

Through the adjustment of airborne IMU angle during initial assembly, the reference 0° direction of the platform is considered consistent with the 0° direction of the UAV, but with the platform translation available, the 0° directions of platform might deflect. In this case, the 0° lines of the platform will move and/or rotate. Among all types of rotation, only the transition along the indication line has a negligible influence on localization accuracy. The non-parallel translation and rotation in any other direction, including the rotation around the zero line, will have a great influence on localization result. When the deflection angle is non-zero, the resolving based on the pitch and azimuth angles output by the platform is actually inaccurate [29]. That is, an error exists in the obtained target relative to the reference direction. For example, you think your eyes are 30° to the left in front of your body, but actually it is 29°. At this time, you need to have an error compensation factor (1°) to correct that. The transformation from the platform coordinate system P to the UAV coordinate system B is shown in Figure 5. 

To determine the transformation relation, the direct relative angular displacements Δ1 and Δ2 between platform and UAV must be determined first. For the concrete method, see the subsequent system description. Since this is a transformation from platform to UAV, converting the determined angular displacements directly into the UAV coordinate system can effectively reduce the workload. Then the angular displacements can be expressed as Δα, Δβ, Δγ and the transformation relation expressed as:

(4) $${\left[x_{b}\;y_{b}\;z_{b}\;1\right]}^{T}=Q_{bp}{\left[x_{p}\;y_{p}\;z_{p}\;1\right]}^{T}=R\times Q_{bp}Q_{pc}{\left[0\;0\;f\;1\right]}^{T},$$

(5) $$Q_{bp}=\left[\begin{array}{cccc} \cos\Delta \alpha & 0 & \sin\Delta \alpha & 0\\ 0 & 1 & 0 & 0\\ -\sin\Delta \alpha & 0 & \cos\Delta \alpha & 0\\ 0 & 0 & 0 & 1 \end{array}\right]\left[\begin{array}{cccc} 1 & 0 & 0 & 0\\ 0 & \cos\Delta \beta & -\sin\Delta \beta & 0\\ 0 & \sin\Delta \beta & \cos\Delta \beta & 0\\ 0 & 0 & 0 & 1 \end{array}\right]\left[\begin{array}{cccc} \cos\Delta \gamma & -\sin\Delta \gamma & 0 & 0\\ \sin\Delta \gamma & \cos\Delta \gamma & 0 & 0\\ 0 & 0 & 1 & 0\\ 0 & 0 & 0 & 1 \end{array}\right],$$

where *Q_bp_* is the conversion matrix from the platform coordinate system P to the UAV coordinate system B.

### 2.3. Transformation from UAV Coordinate System B to UAV Geographic Coordinate System V

**Figure 6:**
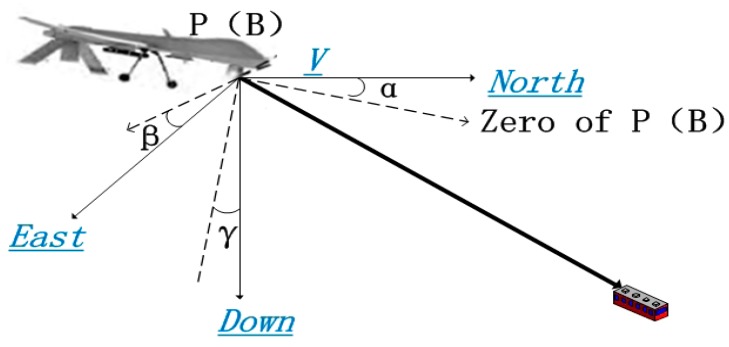
Relationship between UAV coordinate system B to UAV geographic coordinate system V.

The relationship between UAV coordinate system B and UAV geographic coordinate system V is illustrated in Figure 6. 

The UAV geographic coordinate system V in this paper is defined as (North-East-Down) NED, of which the three axes are North Pole, due east and the earth’s core respectively. The transformation relation in this process is:

(6) $${\left[x_{v}\;y_{v}\;z_{v}\;1\right]}^{T}=Q_{vb}{\left[x_{b}\;y_{b}\;z_{b}\;1\right]}^{T},$$

(7) $$Q_{vb}=\left[\begin{array}{cccc} \cos\gamma & -\sin\gamma & 0 & 0\\ \sin\gamma & \cos\gamma & 0 & 0\\ 0 & 0 & 1 & 0\\ 0 & 0 & 0 & 1 \end{array}\right]\left[\begin{array}{cccc} 1 & 0 & 0 & 0\\ 0 & \cos\beta & -\sin\beta & 0\\ 0 & \sin\beta & \cos\beta & 0\\ 0 & 0 & 0 & 1 \end{array}\right]\left[\begin{array}{cccc} \cos\alpha & 0 & -\sin\alpha & 0\\ 0 & 1 & 0 & 0\\ \sin\alpha & 0 & \cos\alpha & 0\\ 0 & 0 & 0 & 1 \end{array}\right],$$

where *Q_vb_* is the conversion matrix from the UAV coordinate system B to UAV geographic coordinate system V.

### 2.4. Transformation from UAV Geographic Coordinate System V to ECEF System E

**Figure 7:**
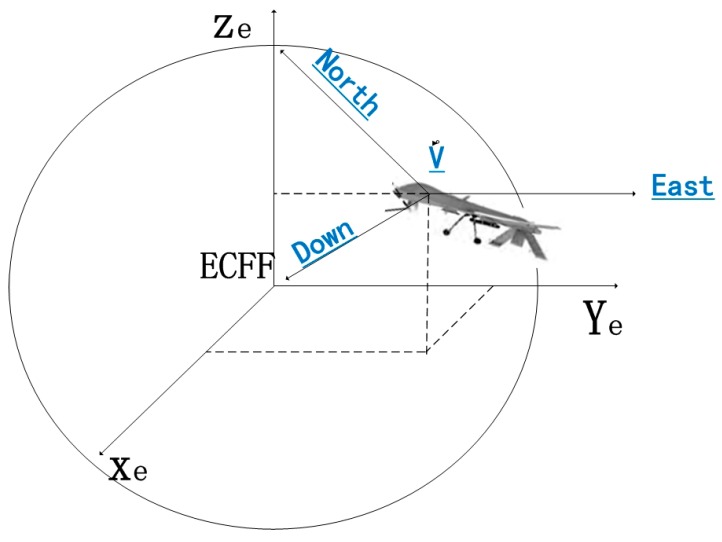
Relationship between UAV geographic coordinate system V and ECEF system E.

The relationship between UAV geographic coordinate system V and ECEF system E is illustrated in Figure 7.

In the ECEF system, the origin is the center of mass of the earth, the axis *z_e_* points from the Earth’s spin axis to the North Pole, the axis *x_e_* points to the intersection between prime meridian and equator, and all the three axes, namely *y_e_*, *x_e_* and *z_e_*, jointly constitute a right-handed coordinate system. The transformation relation in this process is:

(8) $${\left[x_{e}\;y_{e}\;z_{e}\;1\right]}^{T}=Q_{ev}{\left[x_{v}\;y_{v}\;z_{v}\;1\right]}^{T},$$

(9) $$Q_{ev}=\left[\begin{array}{cccc} \cos\gamma & -\sin\gamma & 0 & 0\\ \sin\gamma & \cos\gamma & 0 & 0\\ 0 & 0 & 1 & 0\\ 0 & 0 & 0 & 1 \end{array}\right]\left[\begin{array}{cccc} 1 & 0 & 0 & 0\\ 0 & 1 & 0 & 0\\ 0 & 0 & 1 & \left(N+h\right)\\ 0 & 0 & 0 & 1 \end{array}\right]\left[\begin{array}{cccc} \cos\alpha & 0 & -\sin\alpha & 0\\ 0 & 1 & 0 & 0\\ \sin\alpha & 0 & \cos\alpha & 0\\ 0 & 0 & 0 & 1 \end{array}\right],$$

where *Q_ev_* is the conversion matrix from the UAV geographic coordinate system V to the ECEF system E, and N is the radius of curvature in the prime vertical of the Earth. The semi-major axis of ellipsoid is a = 6,378,137 m, the semi-minor axis is b = 6,356,752 m, the first eccentricity of spheroid is:

(10) $$e=\frac{\sqrt{a^{2}-b^{2}}}{a},$$

and the second eccentricity of the spheroid is:

(11) $$e^{\prime }=\frac{\sqrt{a^{2}-b^{2}}}{b},$$

By combining them with the current latitude, we can obtain:

(12) $$N=\frac{a}{\sqrt{1-e_{e}^{2}\sin^{2}M}}.$$

### 2.5. Transformation from ECEF Frame E to Geodetic Coordinate System G

**Figure 8:**
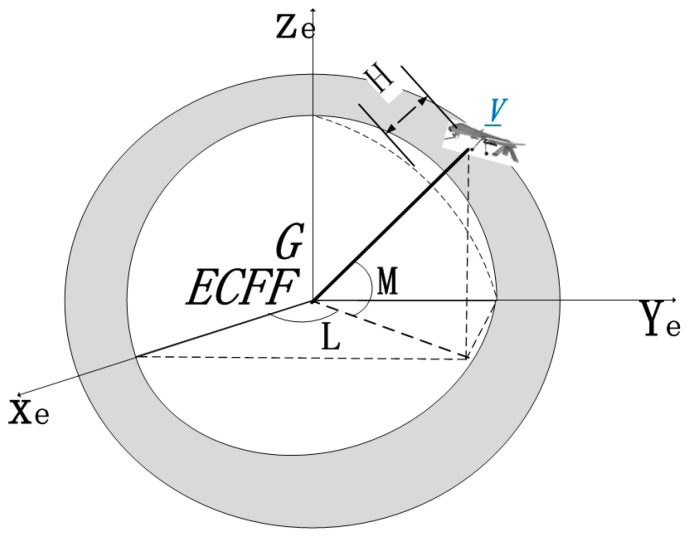
Relationship between ECEF system E and geodetic coordinate system G.

The relationship between the ECEF frame E and geodetic coordinate system G is shown in Figure 8. 

The geodetic coordinate system is spherical. In this frame, the origin is also the center of mass of the Earth, the axis *z_e_* points from the Earth's spin axis to the North Pole, the axis *x_e_* points to the intersection between prime meridian and equator, and all the three axes, namely *y_e_*, *x_e_* and *z_e_*, jointly constitute a right-handed coordinate system. The transformation relation in this process is:

(13) $${\left[x_{g}\;y_{g}\;z_{g}\;1\right]}^{T}=Q_{ge}{\left[x_{e}\;y_{e}\;z_{e}\;1\right]}^{T}+{\left[0\;0\;Ne^{2}\sin M\;0\right]}^{T}$$

(14) $$Q_{ge}=\left[\begin{array}{cccc} 1 & 0 & 0 & 0\\ 0 & \cos M & -\sin\gamma & 0\\ 0 & \sin\gamma & \cos\gamma & 0\\ 0 & 0 & 0 & 1 \end{array}\right]\left[\begin{array}{cccc} \cos L & 0 & -\sin L & 0\\ 0 & 1 & 0 & 0\\ \sin L & 0 & \cos L & 0\\ 0 & 0 & 0 & 1 \end{array}\right]\left[\begin{array}{cccc} 1 & 0 & 0 & 0\\ 0 & 1 & 0 & 0\\ 0 & 0 & 1 & \left(N+h\right)\\ 0 & 0 & 0 & 1 \end{array}\right]$$

where *Q_ge_* is the conversion matrix from the ECFF system E to the geodetic coordinate system G.

A simpler calculation method is to obtain the ECEF system parameters at first, and then to calculate directly various parameters of geodetic coordinate system by using the following equation:

(15) $$\{\begin{array}{c} M=\arctan(\frac{z_{e}+b{e^{\prime }}^{2}\sin^{3}U}{\sqrt{x_{e}^{2}+y_{e}^{2}}-ae^{2}\cos^{3}U})\\ L=\arctan\left(\frac{y_{e}}{x_{e}}\right)\\ H=\frac{\sqrt{x_{e}^{2}+y_{e}^{2}}}{\cos M}-\frac{a}{\sqrt{1-e^{2}\sin^{2}M}} \end{array},$$

(16) $$U=\arctan(\frac{az_{e}}{b\sqrt{x_{e}^{2}+y_{e}^{2}}}).$$

Thus, the target position (M, L, H) in the geodetic coordinate system G can be obtained. During the transformation, different kinds of errors are possible, which are summarized in Table 1. 

These errors have been studied by many scholars [18,30]. This paper only discusses the angular displacements between the platform and UAV, while processing other errors generally in the subsequent simulation. 

## 3. System Composition and Working Principle 

In this paper, the method to correct the relative angular displacement between the platform and the carrier UAV is to separate the platform coordinate system and the UAV coordinate system by adding a transformation matrix to reduce the angular displacement arising from the inconsistent deformation in shock absorber. Decomposing and superposing the angular displacements to the attitude angles of the UAV on the basis of the UAV can reduce the error in coordinate conversion and thus improve the localization accuracy [6].

In order to reduce the hardware requirements on the UAV, this paper assumes that the calculation is carried out by a command station located on the ground. The plane just needs to give its own position and the angle of the target, and the system designed in this paper is only used to measure the amount of angular displacement between the platform and the UAV. The localization process (including the coordinate transformation process) is carried out on the ground through the received data sent by the UAV. The advantage is that on the one hand the hardware consumption of the UAV much less, we can reduce the load weight; on the other hand, computers on the ground can achieve higher accuracy and faster speed.

### 3.1. System Composition 

**Figure 9:**
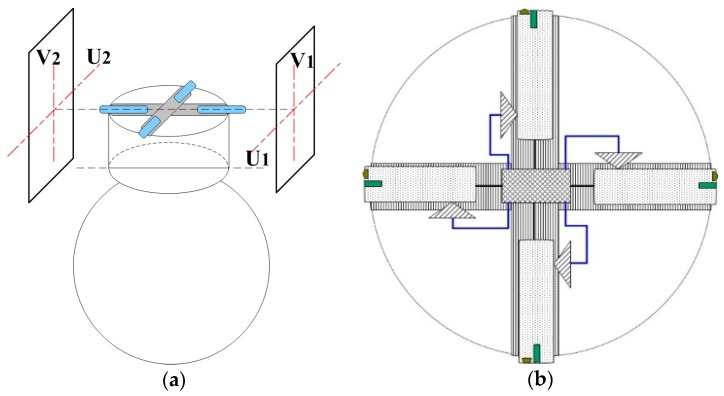
(**a**) System installation position; (**b**) Working principle diagram, top view.

A combination of image registration and coordinate transformation is used to obtain the deflection angle of the platform relative to the UAV. The schematic diagram of the system is shown in Figure 9. For convenience of reading, only the front and rear bulkheads are shown, while the left and right bulkheads are omitted.

The system for correcting the relative angular displacements between the UAV and its onboard platform includes a cruciform main frame, movable probes in the frame, a LED lighting system and a CMOS high-speed imaging system on each probe, a position measurement system, and an information processing system on the central PCB board. The cross frame is fixed on the top of the airborne platform base through a rigid connection, and its center is a PCB circuit board for processing information in real time.

Each probe is in the same plane and each of the outer ends of each probe is provided with an LED illumination system and a CMOS imaging system. Since the bulkhead is semi-closed and dimmed, the LED is used to illuminate the bulkhead area at which each probe is aimed. The imaging system is used for real-time high-frequency imaging of the bulkhead area.

The position measuring system is used for measuring the probe position in each arm and sending the position information to the information processing system, which calculates the total length of each arm according to the probe position information sent by position measurement system and at the same time, carries out registration and comparison with the initial image texture according to a series of bulkhead images obtained by the imaging system. After that, the displacement vectors in the image after registration could be obtained by comparing each center of the two images that has been encoded in a special way. The displacement of the probe relative to the initial position in the plane of the bulkhead can be obtained by a scale calculated following.

Through comprehensive analysis, the angular displacement of the cruciform frame relative to the UAV can be determined. Because the frame and the platform are rigidly connected, this angular displacement can be considered as the angular displacement of the platform relative to the UAV. In the subsequent coordinate conversion, the platform coordinate frame P is separated from the UAV coordinate system B, and a rotation matrix of the platform relative to the UAV is added. The coefficient of this matrix corrects the directional error of the target, thus improving the accuracy of target localization.

### 3.2. The Specific Work Process 

#### 3.2.1. Step 1: Calibrate the Initial Position of the Probes

The pattern is pre-printed on the four bulkheads of the payload capsule. When the zero installation line of the airborne platform coincides with the zero attitude angle of the UAV through adjustment, we fix the platform to the UAV and lift or lower it to its actual working position. Let the center of the frame vertically coincide with the geometric center of the polygon consisting of the connection lines of shock absorbers, and then fix the cruciform frame to the platform base. As the IMU and the platform have the same zero-line direction, it can be considered that there is only a parallel translation between platform coordinate system P and UAV coordinate system B at this time. This translation can be measured on the ground through side projection, but can be left out of consideration considering that the installation position of IMU is generally close to the platform, with only a small impact (on the order of cm) on final localization result. The probe was adjusted to be close to the four sides of the bulkhead and maintain a certain distance. The distance between the front and rear probes is denoted as *l*_12_, and the distance between the left and right probes is *l*_34_. Four illumination systems are then lit to illuminate the bulkhead walls that the probe is facing accordingly. The four CMOS imaging systems collect the image texture of the bulkhead walls and store them in the flash memory on the PCB. The four images at this time are referred to as reference images. Observe the top view of the UAV along the flight direction. The front and rear probes are defined as the front and rear probes, and the left and right probes are defined as the left probe and the right probe respectively. Take the center of the reference image of each bulkhead wall corresponding to each probe as the origin of a coordinate system, the vertical axis as the y axis, and the horizontal direction as the x axis. Four 2D rectangular coordinate systems are set on the plane of bulkhead walls, defined as *x*_1_*O*_1_*y*_1_, *x*_2_*O*_2_*y*_2_, *x*_3_*O*_3_*y*_3_ and *x*_4_*O*_4_*y*_4_. The significance of the bulkhead coordinate system is that the transformation matrix is obtained by differential calculation. And the bulkhead coordinate system does not appear in the subsequent coordinate transformation.

#### 3.2.2. Step 2: Real Time Acquisition and Analysis of Displacement

During the flight, due to the effect of turning, bumps, resistance and other factors, the platform and the cross frame fixed on it would be displaced relative to the UAV. Each CMOS imaging system takes photos for the corresponding bulkhead wall at high frequency. The DSP chip in the information processing system matches and compares the texture of the current image with that of the reference image, and gives the displacement vector (in pixels) of the current image center relative to its original position. After that, we can obtain the displacement vector of every probe projection in the wall plane relative to its own reference origin through conversion using a factor *λ*. By combining the obtained displacement vector with the length of probe arm, the angular displacements of the frame relative to the UAV can be determined. The angular displacements include the displacements of pitch angle, roll angle and azimuth angle.

#### 3.2.3. Step 3: Error Correction

The angular displacement of the frame relative to the UAV, as obtained by Step 2, will be transmitted to the receiver on the ground along with other date. The transformation matrix *Q_bp_* is generated. Error correction is carried out during the localization process.

## 4. Validity Analysis

### 4.1. The Shock Absorber

**Figure 10:**
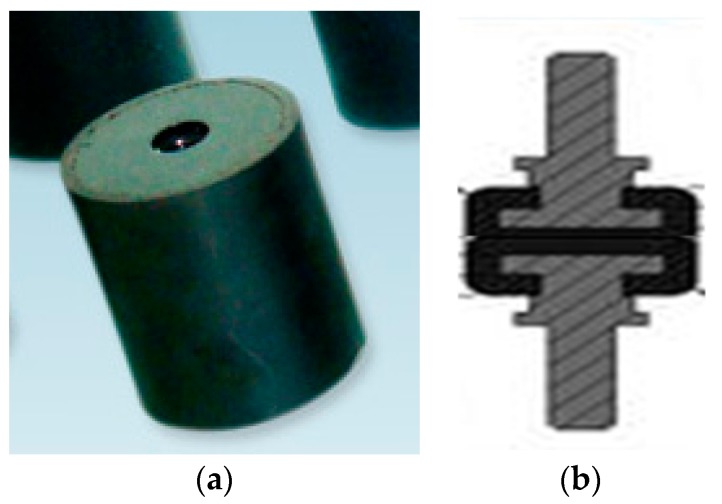
(**a**) Real shock absorber; (**b**) Structure of the shock absorber.

**Figure 11:**
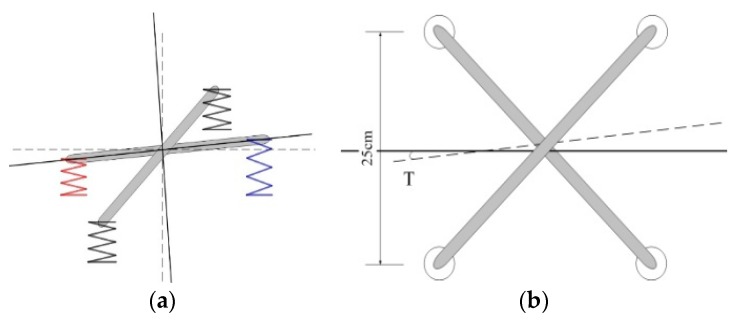
(**a**) Side view of the shock absorber deformation; (**b**) top view of the shock absorber deformation.

The shock absorber used is shown in Figure 10. The black part is made from rubber. Figure 11 shows a side and top view of the displacement of the shock absorber. The radius of the model shock absorber is 1.25 cm. Its deformation in a plane will not exceed its radius, otherwise the shock absorber has been damaged. Here we take the limit value of 1 cm. As the platform is fixed to four shock absorbers, the final angular displacement T will not be large. The square edge of the stent structure is 25 cm. After projection in the plane, with $\left|X_{1}\right|$ ≤ 1 cm and $\left|X_{2}\right|$ ≤ 1 cm, the limit value of angular displacement:

(17) $$\theta_{\max}=\arctan\frac{\left|X_{1}\right|+\left|X_{2}\right|}{L}\leq 4.57{}^{\circ}.$$

Of course, this is a kind of very extreme situation. With the four shock absorbers working together, each single shock absorber will not deform to such a large extent.

**Figure 12:**
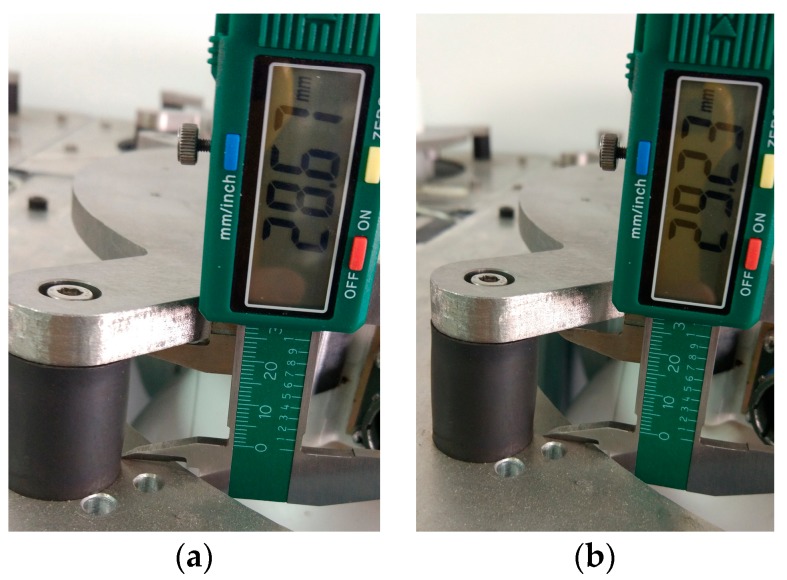
(**a**) Length of absorber before inclination; (**b**) Length of absorber after inclination.

After the introduction, verify the deformation and angular displacement. Figure 12 shows just the change in the length of absorber by comparing the length before and after the platform is placed on an inclined plane. It shows that there is indeed deformation.

The change in length has been 0.56 mm can be measured with the inclination angle of the inclined plane is merely 5°. According to the settings above (25 cm), the angular displacement can reach 0.1283°. It is easy to deduce that an angular displacement of 0.1° is common during the flight.

### 4.2. Image Registration

In the practical work, the image taken by the CMOS imaging system is sent to the information processing system. The DSP chip series [31] C64XX and SIFT algorithm [32] are used to calculate the number of pixels that shift between the reference image and the real-time image. Then, according to the distance between the imaging system and the bulkhead, the factor *λ* between the pixel and the actual distance is calculated. 

**Figure 13:**
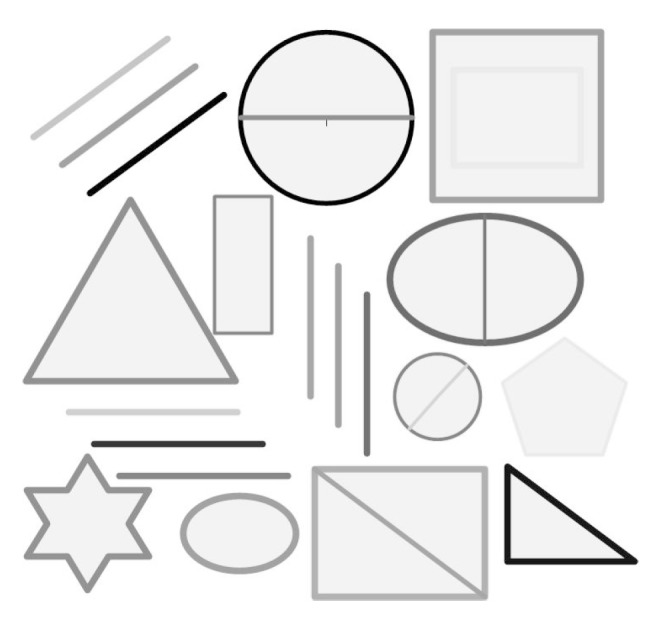
Pre-designed patterns.

As we adopt the pre-designed pattern, it is possible to control the size and kind of the pattern on the bulkhead. In view of the fact that the deformation of the shock absorber is not so great, a pattern having a size of 5 cm × 5 cm is enough. As the lens is close to the bulkhead, the distortion near the edge of the image caused by perspective projection will be large, so a small area in the center of the image is selected as the effective region to match, which can improve the registration speed and accuracy. Paste or print it on the area facing the probe on the bulkhead. We choose a pattern with simple feature, such as a variety of common graphics and directions inconsistent stripes (see Figure 13) to improve the efficiency of registration. 

The pattern can also ensure that a small area has a unique registration result, not mistakenly registered to other areas (for example, you would not match a small area that included both acute and right angles at the same time to another area that include both round and right angles), so it can ensure that any small area has a unique registration result.

### 4.3. The Transformation Matrix

**Figure 14:**
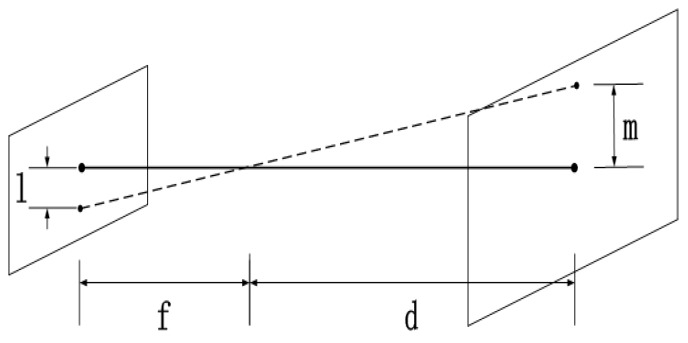
Schematic diagram of pixel and actual displacement.

By image registration, the offset is actually given in pixels. A scale factor *λ* must be converted to be the actual displacement *m* on the bulkhead. A schematic diagram of the pixel and actual displacement are shown in Figure 14. The conversion formula is:

(18) $$\frac{l}{m}=\frac{f}{d}$$

where *l* is the distance on the CMOS sensor, *m* is the actual displacement size on the bulkhead, *f* is the focal length, *d* is the size of the lens on the probe from the bulkhead. 

Take the pixel size of 5 μm as an example. If the focal length of the lens is 10 mm and the lens is 50 mm away from the wall, then the offset of a pixel corresponds to a displacement of 25 μm. That means the actual displacement is:

*m* = 25 μm × *n* (19)

where *n* is the number of pixels offset.

**Figure 15:**
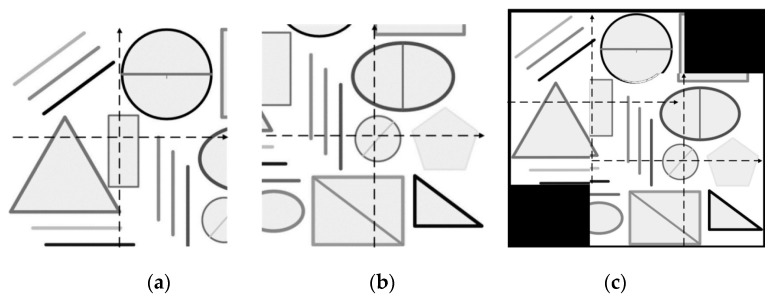
(**a**) Reference image; (**b**) displaced image; (**c**) image after registration.

Subsequent image offset calculation can continue to follow this scale factor. Then as long as the accuracy of image registration is 4 pixels, you can measure a displacement of 0.1 mm. The initial captured image is saved as a reference image, with its image center as the origin of the coordinates, after taking another image with displacement, the two images can be registered, we can see an offset has occurred in the image. This means that the lens has moved relative to its original position. A schematic diagram is illustrated in Figure 15.

When the imaging system moves in the direction of the probe, the real-time image is not on the same scale as the reference image. The registration process would adjust the image after zooming and rotation, and the final result refer to the reference image according to the principle of Scale Invariant Feature Transform (SIFT). Extracting the shape feature and scale in the moving image as invariants can ensure that the pixel-to-real distance scaling relationship is always valid [33].

After obtaining the displacement vector of the probe projecting in the plane of the bulkhead, the differential calculation is carried out to obtain the compensation matrix for correcting the error.

**Figure 16:**
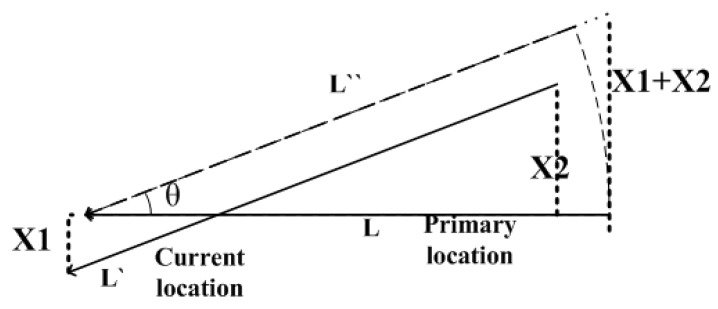
Schematic diagram of actual displacement conversion.

As defined above, the arm length between front probe and rear one is *l*_12_, and the arm length between the left and right probe is *l*_34_. The schematic for calculation of angular displacement size is shown in Figure 16. Pitch angle error is:

(20) $$\Delta \alpha =\arctan\frac{x_{1}-x_{2}}{l_{12}}.$$

Using the same method, we can calculate the roll angle error:

(21) $$\Delta \beta =\arctan\frac{x_{3}-x_{4}}{l_{34}},$$

and yaw angle error:

(22) $$\Delta \gamma =\arctan\frac{y_{1}-y_{2}}{l_{12}}.$$

The transformation matrix used to compensate the offset angular displacement is then obtained:

(23) $$Q_{bp}=\left[\begin{array}{cccc} \cos\Delta \alpha & 0 & \sin\Delta \alpha & 0\\ 0 & 1 & 0 & 0\\ -\sin\Delta \alpha & 0 & \cos\Delta \alpha & 0\\ 0 & 0 & 0 & 1 \end{array}\right]\left[\begin{array}{cccc} 1 & 0 & 0 & 0\\ 0 & \cos\Delta \beta & -\sin\Delta \beta & 0\\ 0 & \sin\Delta \beta & \cos\Delta \beta & 0\\ 0 & 0 & 0 & 1 \end{array}\right]\left[\begin{array}{cccc} \cos\Delta \gamma & -\sin\Delta \gamma & 0 & 0\\ \sin\Delta \gamma & \cos\Delta \gamma & 0 & 0\\ 0 & 0 & 1 & 0\\ 0 & 0 & 0 & 1 \end{array}\right].$$

## 5. Experiments

### 5.1. The System 

**Figure 17:**
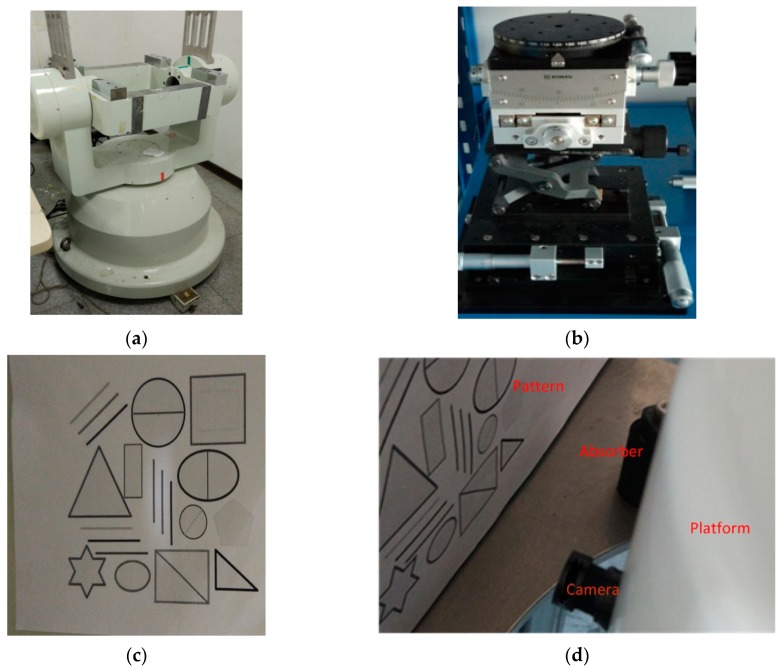

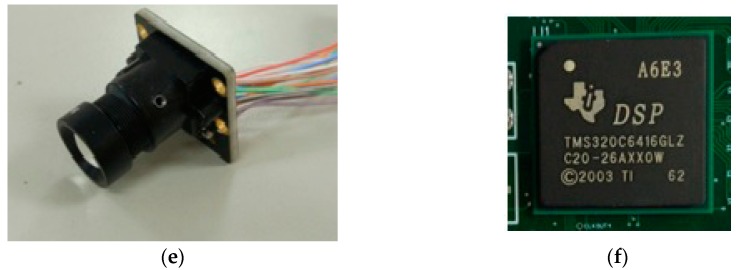
(**a**) the swing table; (**b**) turntable; (**c**) the pattern picture pasted on the swing table; (**d**) position of each part; (**e**) the camera; (**f**) the DSP chip C64XX.

Limited to the experimental conditions and funding, this paper verified the system through a simulation of an actual flight in laboratory. The platform was fixed on the swing table shown in Figure 17, simulating the attitude change during flight. 

We use the multi-direction measuring turntable to measure angles under various attitudes After the vertical axis of the platform is tilted at various small angles with the horizontal plane, the error due to the deformation of the shock absorber can be measured because the center of gravity of the platform is far away from the base part in the vertical direction. The angle can reach 0.1° in the vertical direction. But the pitch angle can be set 0.05° for the upper limit value in the horizontal direction due to tangential deformation is small. Those parameters are set as the original error without optimization. According to the description above, the result of image registration will affect the measurement precision of probe displacement vector. At the same time, the registration time should be as short as possible to meet the real-time requirements [34]. In this paper, due to the use of a simple pattern, the registration algorithm used to be verified time-consuming in the 100-ms level. With the laser range finder limited in the level of several Hz, the solution can be considered real-time calculated. And by using the previous printed pattern, the image registration accuracy can reach the level of 2 pixels. According to above, 2 pixels correspond to the displacement of 0.05 mm.That means that we can measure an error of 0.01146°. That is, the measurement accuracy can be 42’’. Compared with the original 0.1° error, the accuracy has been improved a lot.

### 5.2. Simulation 

Simulation has been carried out in the localization process to prove it can improve the localization accuracy effectively. We used Monte Carlo method to simulate and analyze the target localization accuracy by using a simplified model. Taking into account the actual localization process, parameters that measured by the other sensors onboard are also with errors. So this simulation does not get rid of these errors except for the reference value. The parameters of each section are shown in Table 2. The errors were generated by the standard normal distribution function.

**Figure 18:**
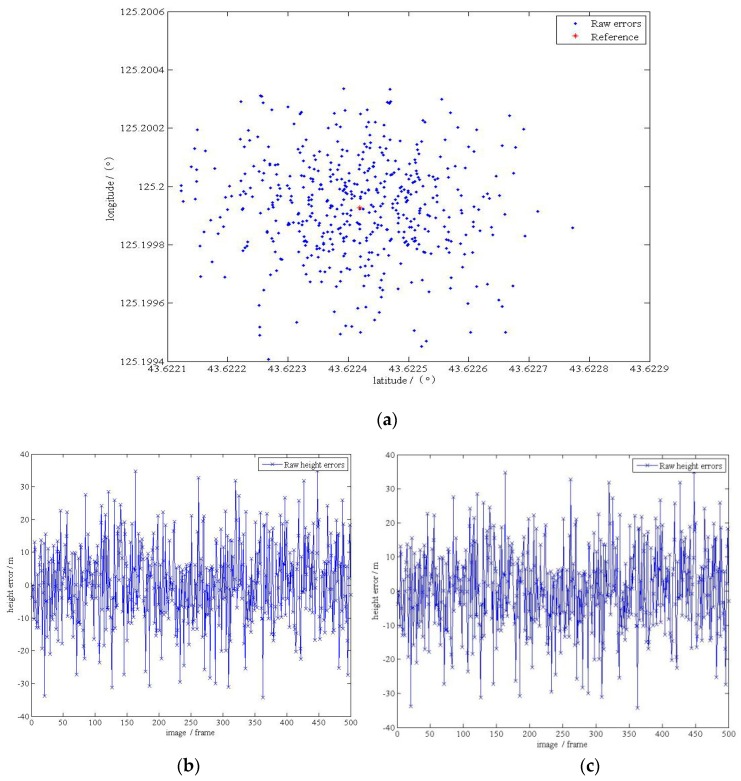
(**a**) The localization results in the plane without angular displacement errors; (**b**) the localization errors in the plane; (**c**) the localization errors in the elevation.

At first, the angular displacement errors presented in this paper were not considered. The Monte Carlo method was used to generate 500 sets of localization parameters at random. The localization results are shown in Table 3 and Figure 18. The reference point was obtained with no angular displacement (set errors as 0°). In order to reduce occasionality, we generate 500 sets of data and obtain the Root Mean Square Errors (RMSE) as the final result. Note that the process was done on the computer, because this system only needs to output a specific angular displacement, which conforms to the actual use of the entire UAV system environment. As mentioned earlier, the use of the computer on the ground will be faster and more accurate.

**Figure 19:**
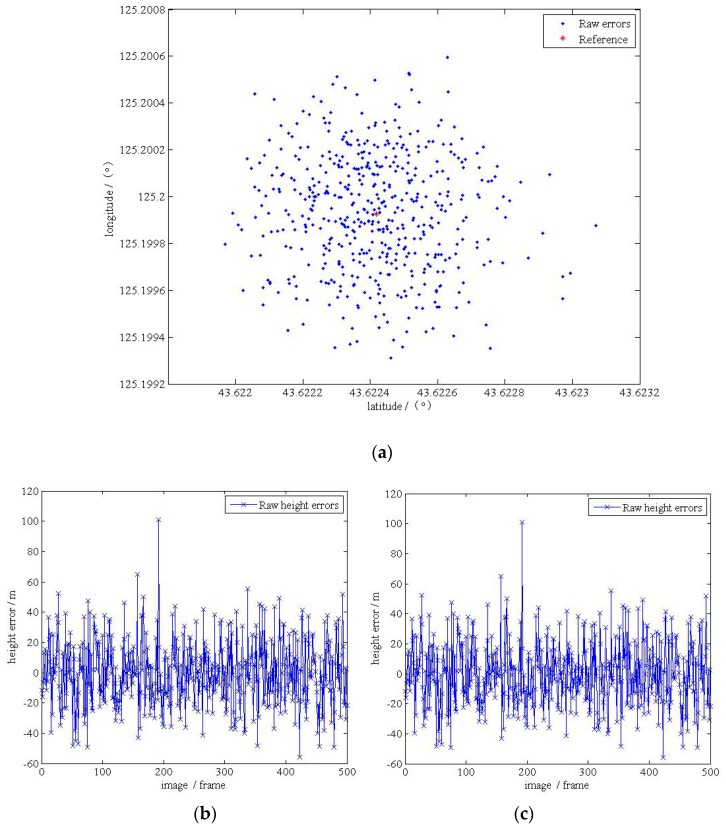
(**a**) The localization results in the plane with initial angular displacement errors; (**b**) the localization errors in the plane; (**c**) the localization errors in the elevation.

When taking the angular displacement errors shown in Table 4 between the platform and the carrier into account, the accuracy significantly decreased with the same other parameters. Results are shown in the Table 5 and Figure 19.

This is the case of a large angle to the horizontal direction. The angular displacement would impact greater if the angle is smaller [3]. The influence of angular displacement on the final localization results could be understood by comparison. When the UAV hovers above the target to reconnoiter it, the roll angle of UAV would be much larger. At this time, different grade deformations of shock absorber would greatly increase the inconsistency of the four distances and the center of gravity of platform is offset with the center of form. As a result, the angular displacement would be much greater than 0.1°. We adopted a conservative value when we carried out simulation in this paper. After correction of relative angular displacements between an UAV and its platform, we can obtain a series of more accurate localization results. As illustrated above, we can measure an error of 0.0115°, so we can set the angle errors to be 0.0115°, as shown in Table 6. 

**Figure 20:**
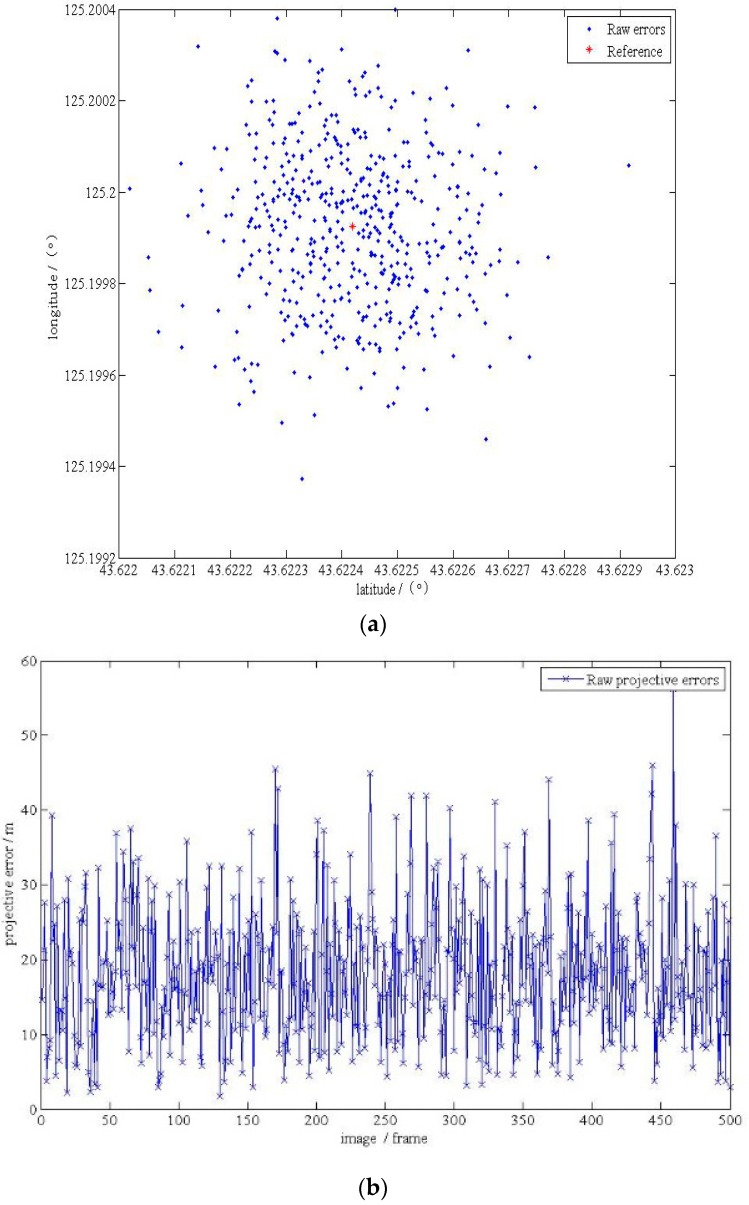

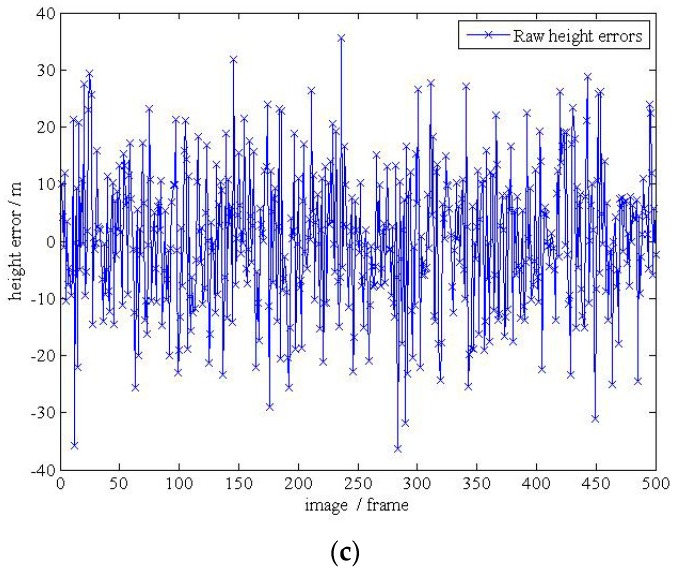
(**a**) The localization results in the plane with correction; (**b**) the localization errors in the plane; (**c**) the localization errors in the elevation.

Results are shown in the Table 7 and Figure 20.

We can obtain significant improvements by comparing the results. The platform was assumed to have an elevation angle of −40° (this elevation angle means the one that LOS of the platform relative to the target) in the simulation above. The influence of angular displacement on the final results can be seen by comparison. When the UAV reconnoiters target at a small elevation angle (set 0° in the horizontal plane), the target is almost in the front of the UAV. In this case, the directional errors—the angular errors—affect the final localization accuracy with a more significant factor. 

Then we carried another simulation to analyze the effect of the system at different angles. We set the elevation angle at −60° to −20° as shown in Table 8 (0° in the horizontal plane) in the case of the remaining parameters of the same with Table 2. After obtaining the results without correction, we compare the results those have been corrected with them. The improvement can be obtained in this way:

(24) $$I=\frac{\left|R-r\right|}{R},$$

where *I* represents the improvement; *R* represents the result without correction and *r* represents the results have been corrected. 

**Figure 21:**
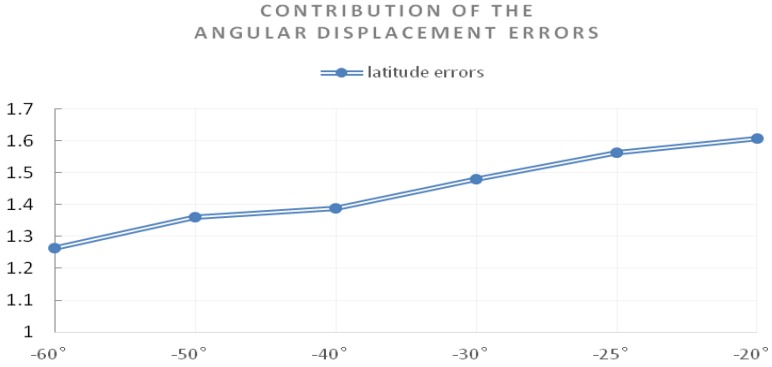
The contribution of the angular displacement errors to final latitude errors.

Figure 21 shows the contribution of the angular displacement errors between the platform and the carrier UAV that have been corrected to final latitude errors (RMSE). The vertical axis represents the final latitude errors with a size of 10^−5^°.

**Figure 22:**
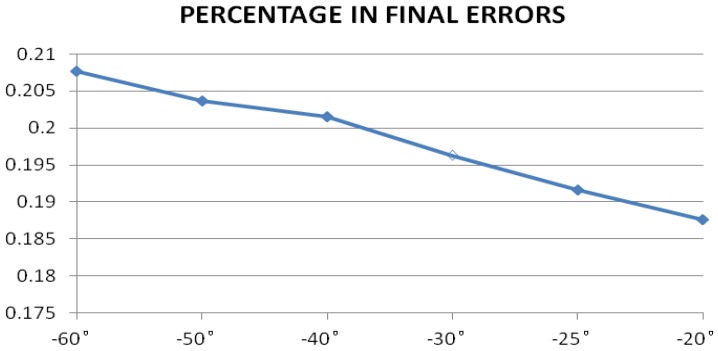
The percentage of the angular displacement errors in final latitude errors.

Figure 22 shows what percentage in the final latitude errors of the angular displacement errors are.

## 6. Discussion

We can conclude from Figure 20 that the effectiveness declines with the decreasing elevation angle, which has been illustrated in Figure 2. The percentage of the corrected errors in the final errors declines as the influence of other directing angle errors on the final result increases. Although the final localization accuracy declines significantly on the whole, we still achieve an improvement in accuracy by 14.34% as shown in Table 8. How to improve the accuracy on the whole with a small elevation angle should be considered in the future.

Simulation results show that the method proposed in this paper can effectively reduce the influence of angular displacement on the final localization results. However, the exploration must continue. Improving the accuracy of the system is still the most important. In this paper, the design of the system still has much room for improvement.

Firstly, in the equation of displacement measurement Equation (17), the resolution of measurement *m* can be smaller by reducing the distance between the probe and the bulkhead walls *d* and increasing the focal length *f* because the size of the pixel size *l* is limited by the CMOS sensor, which may not to be improved in a short period. However, it’s easy to cause the probe to run into the bulkhead walls and get damaged in case of reducing *d*. Increasing the *f* will increasing the cost of the lens sharply. The specific control of d/f ratio should be studied in practice, but must not be blindly reduced. 

Secondly, the registration accuracy of the system can be up to about 2 pixels currently. If the accuracy of a pixel can be improved, the accuracy of the measurement can be doubled. However, in order to meet the requirement of real-time calculation, the registration algorithm cannot be too complex, otherwise the time will be too long and that will increase the burden of calculation, and even result in data flow alternation errors. Taking the 2 Hz laser range finder as an example, this system needs to control the speed of the pattern registration within 500 ms, and better within 200 ms considering the clock signal synchronization, data transmission and other issues. A lot of efforts are still needed to study how to design a better and faster algorithm based on this system or to find the best predesigned pattern in order to improve the registration speed and accuracy.

## 7. Conclusions

The positive effect of the system lies in the fact that through measurement and calculation, a real-time angular displacement is obtained and then can be sent to the receiver, where the resulting angular displacement can serve as the error compensation item to be superposed with the current UAV attitude angle to obtain a more accurate LOS direction helpful for improving the localization accuracy. And this system has a small demand for space and hardware resources. The whole structure is compact, and low-cost, and can be installed in various forms. In terms of chip resources, the software is characterized by small calculation load, high real-time performance and effective correction. Earlier calibration and alignment can be completed in dozens of minutes without adding too much workload. Through the image registration, the displacement in pixels is obtained, which can be proportionally converted into actual displacement, which, in turn, can be used to calculate the error of angular displacement between platform and UAV. With this system, real-time measurement can be taken in flight to improve the single-aircraft single-point localization accuracy, thus laying a good foundation for other improvement methods. 

## Figures and Tables

**Table 1:** Sources of localization error.

Type of Function	Type of Subsystem	Influence Factor
Directional error of photoelectric platform	Optical system	Parallelism and conformance of optical axis
	Mechanical frame	Error in design and installation
	Control system	Error in stabilization and tracking system
	Others	Deformation, vibration, electromagnet interference, wear and tear etc.
Alignment error of photoelectric platform and INS	Installation alignment	Initial directional and horizontal leveling
	Shock absorber	High-frequency angular vibration and low-frequency shaking
Error in UAV (INS) motion parameter	Attitude measurement (INS)	UAV attitude measurement error
	Position measurement	UAV localization error
Range error	Laser range finder	Measurement error of range finder
Coordinate transformation error	Error in the transformations among different geodetic coordinate systems	

**Table 2:** Initial parameters and their error range.

Type	Focus	Laser Range	Azimuth Angle of Platform	Elevation Angle of Platform	Pitch	Roll	Yaw	Longitude	Latitude	Elevation of UAV
Reference	100 mm	12000 m	5°	−40°	0°	0°	0°	125.19°	43.54°	8000 m
Error range	5%	10 m	0.02865°	0.02865°	0.03°	0.03°	0.06°	0.0001°	0.0001°	10 m

**Table 3:** Localization accuracy without angular displacement errors.

Configure	Longitude	Latitude	Elevation
Localization results	125.199925840984° E	43.6224190056630° N	293.18 m
Error (RMSE)	0.000170295986306348°	0.000119554074008099°	12.4911 m

**Table 4:** The angular displacement errors without correction.

Angular Displacement Errors	Pitch	Roll	Yaw
Range	0.1°	0.1°	0.05°

**Table 5:** Results without correction.

Configure	Longitude	Latitude	Elevation
Error (RMSE)	0.000249456444498644°	0.000184640982639463°	21.53 m

**Table 6:** The angular displacement errors with correction.

Angular Displacement Errors	Pitch	Roll	Yaw
Range	0.0115°	0.0115°	0.0115°

**Table 7:** Results with correction.

Configure	Pitch	Roll	Yaw
Error (RMSE)	0.000174500400122541°	0.000132910371952061°	14.3658 m

**Table 8:** Improvement made by the system with different angle.

**Current Angle**	−60°	−50°	−40°	−30°	−25°	−20°
**Improvement**	39.12%	33.24%	31.13%	24.63%	18.56%	14.34%
