# Correction: Liu, C., et al. A Novel System for Correction of Relative Angular Displacement between Airborne Platform and UAV in Target Localization*. Sensors* 2017, *17*, 510

**DOI:** 10.3390/s19173639

**Published:** 2019-08-21

**Authors:** Chenglong Liu, Jinghong Liu, Yueming Song, Huaidan Liang

**Affiliations:** 1 Changchun Institute of Optics, Fine Mechanics and Physics, Chinese Academy of Sciences, Changchun 130033, China; 2 University of Chinese Academy of Sciences, Beijing 100049, China

The authors wish to make the following correction to this paper [1]. Due to inadvertent errors caused by adjusting the resolution, replace:

**Figure 18:**
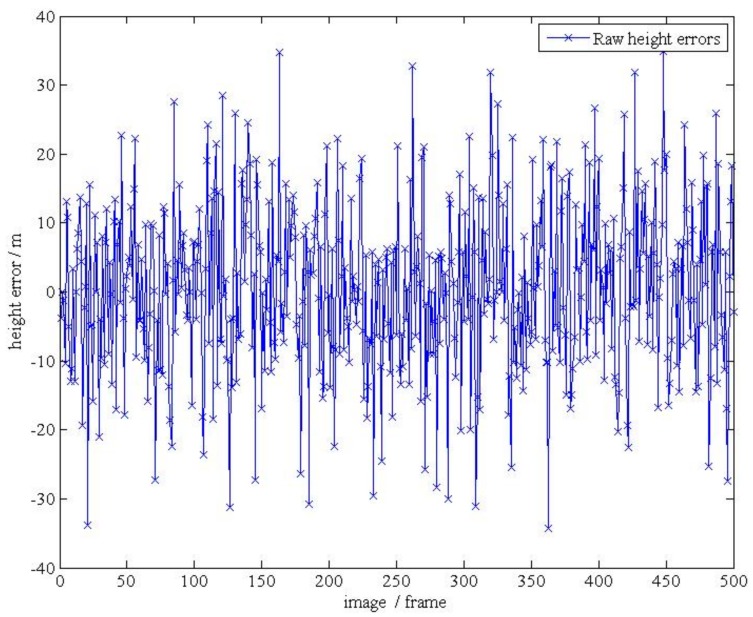
(**b**).

with

**Figure 18:**
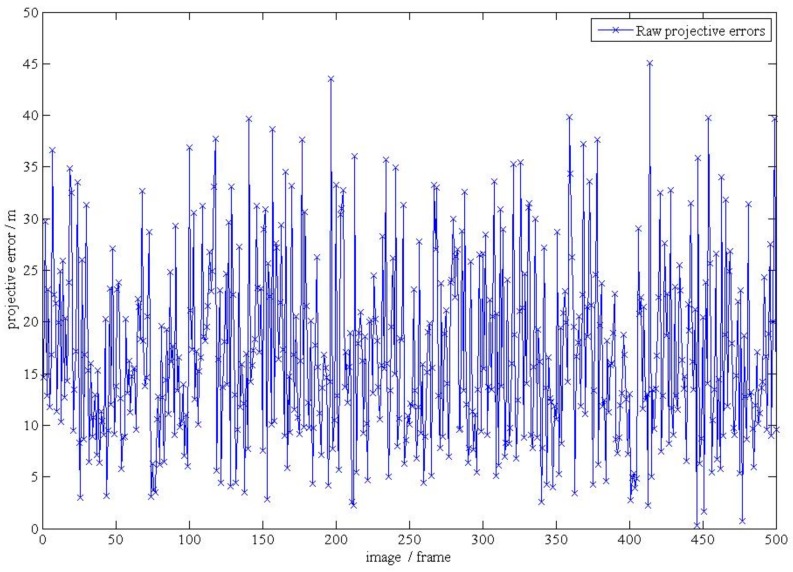
(**b**).

And replace:

**Figure 19:**
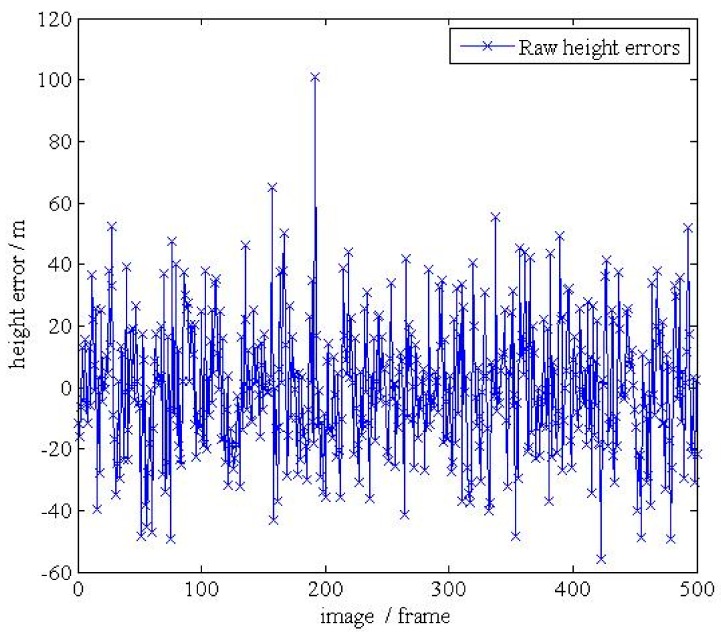
(**b**).

with

**Figure 19:**
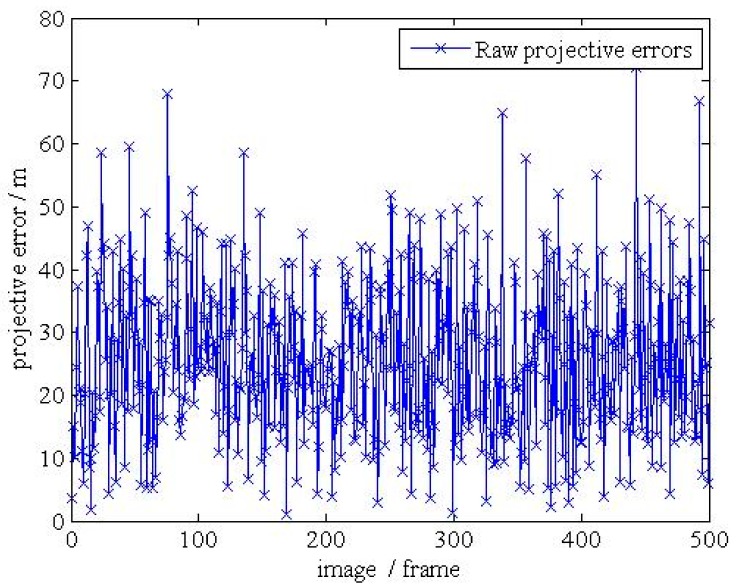
(**b**).

The authors would like to apologize for any inconvenience caused to the readers by these changes.
